# Reduction of Festinating Clinical Gait Parameters to Near Normal After Hip Joint Mobilization: A Parkinson’s Disease Case Report

**DOI:** 10.7759/cureus.88242

**Published:** 2025-07-18

**Authors:** Chistopher K Wong, Chelsea E Macpherson

**Affiliations:** 1 Rehabilitation and Regenerative Medicine, Columbia University Irving Medical Center, New York, USA; 2 Biobehavioral Science, Columbia University Teachers College, New York, USA

**Keywords:** a case study, festinating gait, gait speed, manual therapy physiotherapy, osteopathic manipulative medicine, parkinson’s disease

## Abstract

People with Parkinson's disease (PD) walk with decreased speed and step length that worsen with disease progression. Despite treatments that yield statistically significant yet clinically small changes, normal gait parameters are rarely restored. Few PD studies assess joint passive range-of-motion concurrently with temporospatial gait parameters or joint mobilization effects on gait outcomes. This case introduces hip joint mobilizations for passive range of motion in PD and the unusual outcome of temporospatial gait parameters returning to near normal. A male with PD managed consistently pharmacologically presented with bradykinesia, rigidity, and characteristic festinating gait dysfunctions: slow speed, short step lengths, and fast cadence. Bilateral hip passive range of motion was impaired in all planes. He attended only two sessions for hip joint mobilizations and stretching. Hip passive range of motion increased. Temporospatial gait parameters also improved to near age-sex-matched normal: speed increased 20.4% from 1.08 to 1.30 m/s, well beyond the minimal clinically important difference; step length increased 30.2% from 0.53 to 0.69 m; and cadence decreased 7.3% from 2.05 to 1.90 steps/s. After two weeks, large clinically important intersession improvements remained in temporospatial gait parameters with near-normal gait speed without visible festination. Having returned to tennis, he considered his goals met and declined further care, still playing tennis seven months later. Controlled research exploring relationships among passive range of motion, joint mobilization, and gait parameters in PD is warranted.

## Introduction

People with Parkinson's disease (PD), a progressive multi-system disorder [1], display the cardinal signs of rigidity, bradykinesia, postural instability, and gait dysfunction [2]. Festinating PD gait is characterized by short step lengths and slow walking speed with or without altered cadence and freezing-of-gait [3]. Medically managed pharmacologically, the PD physiotherapy Clinical Practice Guidelines (CPG-PD) strongly recommend multi-modal rehabilitation for intertwined physiologic and musculoskeletal impairments with aerobic, resistance, balance, gait, and task-specific training and weakly recommend stretching based on one study [4]. PD movement dysfunction worsens musculoskeletal impairments, while pre-existing musculoskeletal impairments worsen movement dysfunction apparent in temporospatial gait measures [3]. Despite much exercise and movement training research [4], passive joint motions necessary for active movement, treatments to address joint impairments, and concurrent PD gait assessment remain unexplored [5].

No CPG-PD articles examined passive joint range-of-motion impairments [4], which represent the mechanical limit and potential for active movements powered by muscles and observed in gait analysis studies as limited throughout the lower limb [6]. Basal ganglia-brainstem system dysfunction in PD affects muscle tone, active movement, and locomotion via motor neurons that directly innervate muscles, but not joint capsules [7]. Stretching, which requires underlying joint mobility and passive range of motion, can improve muscle flexibility but not PD temporospatial gait parameters [4]. Joint passive range-of-motion, however, is rarely assessed in PD, with just one study showing passive hip range-of-motion minimally increased after 48 exercise sessions [8]. Spinal manipulative studies all lack passive range-of-motion measurements, and no CPG-PD studies explored joint mobilizations with temporospatial gait outcomes [4,5]. This PD case, previously presented as a poster at the American Physical Therapy Association Combined Sections Meeting in February 2023, introduces hip joint mobilizations aimed at increasing the mechanical range of motion available for gait and the unusual outcome of temporospatial gait parameters improved to near age-sex-matched normal at two weeks with a seven-month follow-up.

## Case presentation

Informed written consent was obtained, confidentiality rights were preserved, and CARE guidelines were followed, though approval from the participating medical center's institutional review board was not required for this case report. One licensed board-certified orthopedic clinical specialist physiotherapist performed all assessments and treatments. All case data were obtained from the electronic medical record.

The 51-year-old male patient was diagnosed with PD three years earlier when bilateral motor symptoms, bradykinesia, rigidity, freezing-of-gait, and intention tremor became apparent. Dopamine transporter single-photon emission computed tomography imaging confirmed the diagnosis. His pharmacological management included consistent carbidopa/levodopa, rotigotine, and amantadine doses. Despite being in an on state, function declined over six months, and worsening festination prevented participation in his weekly tennis games. He denied pain and walked independently without an assistive device. His only physiotherapy goal was to improve gait to enable return to tennis.

Findings

Evaluation revealed flexed posture, bradykinesia, rigidity, and intention tremor without freezing-of-gait. Gait was slow with short shuffling steps clinically assessed over a 40-meter walkway with one 180-degree turn using a digital stopwatch and manually recorded step counts yielding gait speed (distance/time), step length (distance/steps), and cadence (steps/time). Initial temporospatial gait parameters were comparable to men with PD, with slower speed, shorter steps, and faster cadence than non-PD age-sex-matched peers [9,10] (Table 1). Standard goniometry revealed limited hip passive range of motion and end-range accessory joint hypomobility. Manual muscle tests revealed 4/5 hip strength throughout. 

**Table 1:** Case data with age-matched normal values for men with and without Parkinson’s disease. AB: Abduction, E: Extension, ER: External Rotation, ROM: Range-of-Motion * Increase exceeded minimal clinically important difference in Parkinson’s disease.

Outcome Measure		Men with PD^a^	Session 1: pre-treatment	Session 1: post-treatment	Session 2: pre-treatment	Session 2: post-treatment	Men without PD
Bilateral Passive ROM (degrees)	Hip AB	No data	10	25	25	25	39
	Hip E	No data	10	10	10	20	7.5
	Hip ER	No data	30	30	30	50	50 – 55
Gait speed (m/s)		0.87–1.09	1.08	1.14	1.21	1.30 *	1.38-1.49
Step length (m)		0.51-0.63	0.53	0.71	0.60	0.69	0.71
Cadence (steps/s)		1.68–1.73	2.05	1.60	2.00	1.90	1.89

Diagnosis

His presentation aligned with the postural instability/gait dysfunction (PIGD) phenotype and Hoehn-Yahr Stage 3 mild to moderate diagnosis, including bradykinesia, rigidity, postural instability, and gait dysfunction [2]. Prognosis, despite consistent medication, was disease progression and functional decline [2]. Even sustained adherence to weekly physiotherapy typically produces small effect size gait speed changes [4]. Gait speed is the product of cadence and step length, which appears 2-dimensional in sagittal plane double support measurements but results from active hip motion to position the limb in all planes (sagittal-extension, frontal-abduction, and transverse-rotation) [11]. 

Plan of care

The personalized patient-centered physiotherapy care plan focused on reducing gait dysfunction and facilitating lateral frontal plane hip mobility required for return-to-tennis [12,13]. Thus, intervention emphasized maximizing hip joint passive range of motion in all planes to increase active step length potential and gait speed, whether impairment preceded disease onset or resulted from PD muscular rigidity [3]. Since the basal ganglia-brainstem system directly controls muscle rigidity but not joint capsule hypomobility [7], joint mobilizations to optimize passive range of motion were prioritized over muscle flexibility. While people function without full joint range of motion, movement becomes limited and dysfunctional compensations occur, including short step lengths and slow speed that characterize festinating gait [3] (Table 1). Biweekly sessions for eight weeks were planned to reduce joint impairments before progressing to functional exercise and return-to-tennis activities.

Intervention

**Figure 1:**
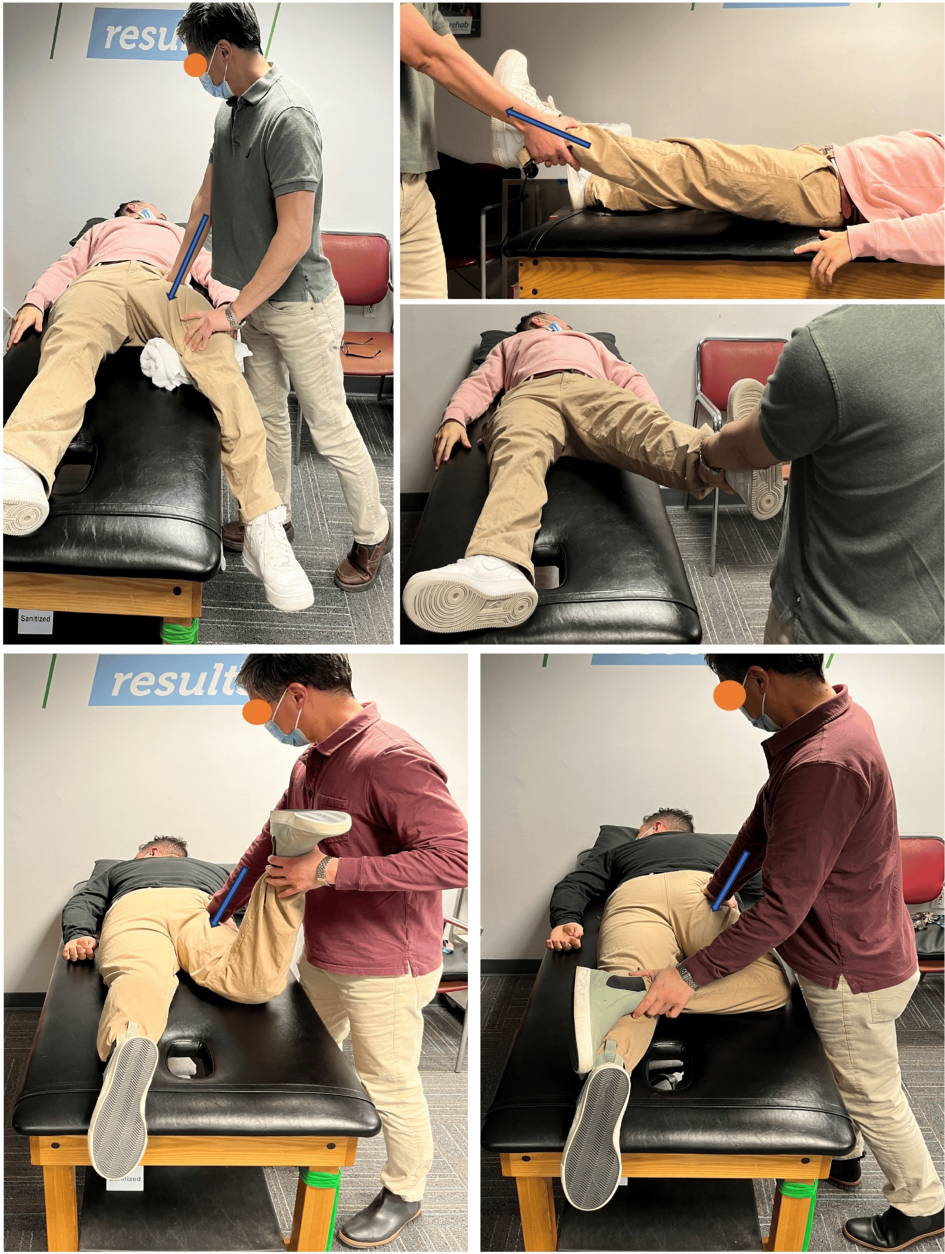
Hip joint mobilizations were performed in the case. Top: session-1 hip joint mobilizations to increase abduction: Left) posteriorly directed grade III+ oscillations in supine with hip in slight flexion and abduction for general mobility biased towards abduction and to prepare for thrust. Right) Long axis distraction grade V thrust modified in slight flexion, internal rotation and abduction for frontal plane impact and hip abduction range of motion. Bottom: Session-2 hip joint mobilizations to increase hip extension and external rotation. Left) Anteriorly directed grade III+ oscillations in the prone knee bend position to increase anterior capsule mobility and rectus femoris muscle flexibility for sagittal plane impact and hip extension range-of-motion. Right) Anteriorly directed grade III+ oscillations in the prone figure-of-four position to increase anterior capsule mobility for transverse plane impact and hip external rotation range-of-motion.

He attended only two sessions, two weeks apart. The session-1 evaluation included treatment constrained to hip joint mobilizations (~15 minutes) [14] and ~15 minutes of home exercise program education, including lunge stretches to maintain step length: 3-5 repetitions, 30s each (Figure 1). The session-2 reassessment revealed that improved hip passive range of motion and gait parameters were maintained (Table 1). Session-2 treatment included hip joint mobilizations (~15 minutes) [14] and home program education (~15 minutes), adding prone figure-of-four position stretching to maintain new hip abduction and external rotation range-of-motion (3-5 repetitions, 30s each) and encouragement to continue daily walking. While comprehensive care was planned, he discontinued physiotherapy two weeks later because his walking and return-to-tennis goals were met.

Outcomes

From first to last assessment, gait speed increased by 20.4% (0.22 m/s), exceeding the 0.06 m/s minimal clinically important difference (MCID) in PD and indicating an upper-range large effect size change [15]. Improvements occurred within- and between-sessions in hip passive range-of-motion, step length, and gait speed, with all approaching age-sex-matched norms [9,10,16] (Table 1). Cadence decreased 7.3%. Step length increased 30.2% (0.16 m), exceeding the ≤0.08 m statistically significant yet small effect size changes from various non-pharmacologic PD treatments [17]. Visible festination was not apparent upon reassessment, for him, an important clinical outcome. He reported immediate walking improvements and considered his walking goals met, and he achieved his primary return-to-tennis goal after two sessions. At the seven-month follow-up, he walked without visible festination and played tennis regularly, so he declined reassessment and further care.

## Discussion

After two sessions emphasizing joint mobilization, this patient had passive hip range-of-motion, gait speed, and step length improvements beyond MCID and common PD treatments [15,17]. Temporospatial gait parameters initially sex-matched for PD were restored to near healthy age-sex-matched norms [9,10,18], a personally significant clinical improvement potentially more impactful than statistically significant small effect size changes. Multiple body systems affect locomotion, but movement is expressed and kinematically measured by musculoskeletal motions. Underlying passive joint hypomobility restricts active musculoskeletal movement and function, and this patient’s 3-plane hip passive range-of-motion impairment restricted gait and tennis-specific lateral movements [3,11,13]. For him, both hip passive range-of-motion and temporospatial signs of festinating gait improved after physiotherapy, emphasizing joint mobilizations, not stretching muscles directly affected by PD basal ganglia-brainstem dysfunction [7].

Multimodal PD rehabilitation includes range-of-motion exercises, yet the clinical practice guideline for Parkinson's disease (CPG-PD) cites none, including joint mobilizations [4]. The three PD spinal manipulative studies lack passive range-of-motion and follow-up temporospatial gait measures [5]. For non-PD neurological diseases, ankle mobilizations show immediate range-of-motion and gait speed improvements [19]. Because ankle, knee, and hip impairments impact gait with distal-to-proximal progression across different PD stages [20], analyzing specific gait-related joint impairments and personalizing PD rehabilitation with targeted joint mobilization may optimize outcomes [12,20].

Case studies cannot determine cause and effect. PD is progressively neurodegenerative despite his consistent pharmacological treatment and independent physical activity before functional decline stopped him from playing tennis, a progression inconsistent with his rapid recovery of near-normal step length and gait speed after two joint mobilization sessions [2]. Gait speed (m/s) increases with longer step lengths (m/step), potentially affected by increased passive hip range-of-motion, assuming similar cadence (step/s) [6]. Mechanisms explaining how mobilizations increase passive joint range-of-motion remain unclear, but passive range-of-motion absent pain does not vary as active range-of-motion does. 

Case studies highlight novel approaches and unusual outcomes, prompting future study. PD research incorporating passive joint range-of-motion assessments with temporospatial gait parameters would clarify the relationship between joint impairments and gait dysfunction [5]. Without concurrent assessment, understanding of PD gait dysfunction is incomplete. The one PD study to assess gait and passive hip joint range of motion in an eight-month multi-modal exercise program found minimal single-plane improvement without functional effects [8]. Exercise and functional tasks are constrained by the available passive range of motion, potentially explaining limited functional benefit [8]. Increasing passive joint range of motion to maximize potential active movement before functional exercise training is a logical treatment sequence, warranting investigation.

All case reports lack randomized controlled conditions. Limitations also included a lack of instrumented gait analysis common in research laboratories, though unusual in private clinics, one clinician performing assessments and treatments reflecting real-life clinical practice, and patient-reported long-term follow-up. Freezing-of-gait never occurred, though festination can be episodic. The patient met his goals quickly and thus did not need or complete planned multi-modal care, though without behavioral change, improvement could be short-lived. The two-week between-session carryover and seven-month walking and tennis outcomes were promising, even if longevity remains unknown.

## Conclusions

Joint stiffness and limited passive hip range of motion have rarely been studied concurrently with temporospatial gait parameters in people with PD. While the short steps of festinating gait in PD are a well-known phenomenon generally attributed to the neurodegenerative disease course and progressive muscle rigidity, joint capsules are not directly innervated by motor neurons and mechanically limit range-of-motion, thus providing an avenue to gain hip range-of-motion and improve step length and thereby gait speed.

In this PD case, temporospatial parameters typical for festinating gait increased by a large effect size to near age-sex-matched normal after two hip joint mobilization and stretching sessions without pharmacologic changes. While various treatments yield small statistically significant changes in PD gait, restoring gait to near-normal without festination is a novel occurrence with a clinical personal impact. Assessing passive hip range-of-motion with temporospatial gait parameters in controlled PD trials of joint mobilization is warranted.
